# DCE-MRI of esophageal carcinoma using star-VIBE compared with conventional 3D-VIBE

**DOI:** 10.1038/s41598-021-03171-5

**Published:** 2021-12-16

**Authors:** He-Ping Deng, Xue-Ming Li, Liu Yang, Yi Wang, Shao-Yu Wang, Peng Zhou, Yu-Jie Lu, Jin Ren, Min Wang

**Affiliations:** 1 Sichuan Cancer Hospital and Institute, Sichuan Cancer Center, School of Medicine, University of Electronic Science and Technology of China, 55# Lan 4 RenMing Road (South), Chengdu, 610041 Sichuan China; 2 Xi’an Branch of Siemens Healthcare Ltd., Xian, 710075 China

**Keywords:** Cancer imaging, Gastrointestinal cancer, Imaging

## Abstract

To investigate the value of the star-VIBE sequence in dynamic contrast-enhanced magnetic resonance imaging of esophageal carcinoma under free breathing conditions. From February 2019 to June 2020, 60 patients with esophageal carcinoma were prospectively enrolled to undergo dynamic contrast-enhanced magnetic resonance imaging (DCE-MRI) with the K-space golden-angle radial stack-of-star acquisition scheme (star-VIBE) sequence (Group A) or conventional 3D volumetric-interpolated breath-hold examination (3D-VIBE) sequence (Group B), completely randomized grouping. The image quality of DCE-MRI was subjectively evaluated at five levels and objectively evaluated according to the image signal-to-noise ratio (SNR) and contrast-noise ratio (CNR). The DCE-MRI parameters of volume transfer constant (Ktrans), rate constant (Kep) and vascular extracellular volume fraction (Ve) were calculated using the standard Tofts double-compartment model in the post-perfusion treatment software TISSUE 4D (Siemens). Each group included 30 randomly selected cases. There was a significant difference in subjective classification between the groups (35.90 vs 25.10, *p* = 0.009). The study showed that both the SNR and CNR of group A were significantly higher than those of group B (*p* = 0.004 and < 0.001, respectively). There was no significant difference in Ktrans, Kep or Ve between the groups (all *p* > 0.05). The star-VIBE sequence can be applied in DCE-MRI examination of esophageal carcinoma, which can provide higher image quality than the conventional 3D-VIBE sequence in the free breathing state.

## Introduction

Esophageal carcinoma is a common malignant tumor of the digestive system with worldwide incidence^1^. The routine imaging methods include ultrasound, X-ray barium meal, computed tomography (CT) and magnetic resonance imaging (MRI). Due to its characteristics of no radiation and good soft tissue resolution, in some previous studies, MRI has been used in the preoperative staging^2^ and efficacy evaluation of radiotherapy and chemotherapy for esophageal carcinoma^3^. DCE-MRI can quantitatively evaluate the microcirculation, microvascular density, drug retention and uptake function of tumor tissues and has shown high value in clinical application^4,5^. Currently, the routine DCE-MRI scanning protocol for esophageal carcinoma is a conventional 3D-VIBE sequence in the state of free breathing. However, the star-VIBE sequence was filled with radial sampling mode, therefore, motion artifacts can be effectively controlled in this sequence^6^. Currently, this sequence has been reported in DCE-MRI of tumors, such as prostate and gastric^7,8^, this study was conducted to explore the value of DCE-MRI examination in esophageal carcinoma with the star-VIBE sequence.

## Material and methods

### Study population

The study was carried out in accordance with the principles of the Declaration of Helsinki. This study was approved by the medical ethics committee of Sichuan Cancer Hospital (approval number: SCCHEC-02-2019-003), and informed consent was obtained from all the subjects. In addition, the approved consent publishing images of cases was acquired from the patients. Sixty patients with esophageal carcinoma confirmed by pathology and without any treatment from February 2019 to June 2020 were prospectively enrolled in this study. The star-VIBE sequence (Group A) and conventional 3D-VIBE sequence (Group B) were randomly selected by the MRI technician. The inclusion criteria were as follows: patients who had clear self-consciousness and could cooperate well with the technician; patients without metal implants in the lesion area; and patients without a history of allergy to MR contrast agent. Finally, group A included 23 males and 7 females, with an average age of 63.3 ± 8.9 years, and group B included 24 males and 6 females, with an average age of 65.3 ± 9.4 years.

### MR imaging

All subjects were examined with a 3.0 T whole-body scanner equipped with an 18-channel body phased front coil and 32-channel spine coil (MAGNETOM Skyra; Siemens Healthcare, Erlangen, Germany) in the supine position at rest. Patients in both groups were trained to breathe evenly and calmly before examination. First, the conventional plain sweep sequence (T_2_ fat suppression coronal and axial, T_1_ axial) and T_1_ WI VIBE fat suppression with flip angles of 2° and 12° were performed in all patients. Then, DCE-MRI was performed in group A with TR 4.12 ms, TE 1.76 ms, FOV 380 mm × 310 mm, slice thickness 3 mm,, averages 1, matrix 154 × 256, measurements 27, pause after measurement 0 s, subframe interval 2, temporal resolution 12.4 s, scanning time 334 s; and group B with TR 3.72 ms, TE 1.32 ms, FOV 380 mm × 310 mm, slice thickness 3 mm, phase oversampling 30%, averages 1, matrix 195 × 320, iPAT: CAIPIRINHA 4, measurements 24, pause after measurement 0 s, temporal resolution 13.4 s, scanning time 321 s. In both groups A and B, 0.4 ml kg^−1^ GD-DTPA (Guangzhou Consun Pharmaceutical Co. Ltd; specification: 15 ml: 7.04 g) was intravenously injected at a speed of 3.0 ml s^−1^ with an injector (Shenzhen Antmed Co. Ltd; Model: Imastar MDP) in the cubital vein.

### Image analysis

The DCE-MRI images were analyzed by one radiologist and one technician with more than 5 years of experience.

### Subjective image quality classification

The images were classified as follows: Level 1: the overall quality was very poor, and the lesion cannot be displayed; Level 2: the overall quality was poor, the lesion was visible but the boundary of the lesion could not be distinguished; Level 3: the image quality was general, the lesion was visible, the boundary part of the lesion was clear, but the internal display was poor; Level 4: the image quality was satisfactory, the lesion was clearly displayed, the edge of the lesion could be displayed, and the inner part of the lesion showed well; and Level 5: the image quality was good, the lesion and the edge of the lesion showed well, and there was no obvious respiratory artifact in the whole image.

### Objective image quality evaluation

The signal intensity of the lesion and transverse process spinous muscle (SI_d_ and SI_m_, respectively) and standard deviation (SD) of the background signal were measured at the maximum level of the lesion on the middle phase of perfusion images in both groups (Group A, phase 14th; Group B, phase 13th). Then, the signal-to-noise ratio (SNR = SI_d_/SD) and contrast-noise ratio (CNR = [SI_d_ − SI_m_]/SD) were calculated.

### Calculation of the perfusion results

The DCE-MRI parameters of Ktrans, Kep and Ve were calculated with the standard Tofts double-compartment model in the post-perfusion treatment software TISSUE 4D (Siemens). The region of interest was delineated in the solid part of the tumor with obvious enhancement, avoiding necrotic tissue, large vessels, bone and esophageal cavity.

### Statistical analysis

The chi-square test was used to analyze whether there was a difference in sex between the two groups; a nonparametric test was used to analyze whether there were differences in image quality classification between the two groups. The age, SNR, CNR and perfusion parameters of Ktrans, Kep and Ve of the groups were analyzed by Student’s t test. All statistical analyses were performed with SPSS 21.0 software (IBM, Armonk, New York, USA), and a two-tailed *p* < 0.05 was considered significant.

## Results

### Baseline characteristics

By random grouping, each group included 30 cases. There was no significant difference in gender between the two groups (χ = 0.098, *p* = 0.754). No significant difference in age was found between groups A and B (63.3 ± 8.9 vs 65.3 ± 9.4, *p* = 0.411).

### Comparison of image quality grading between the groups

**Figure 1:**
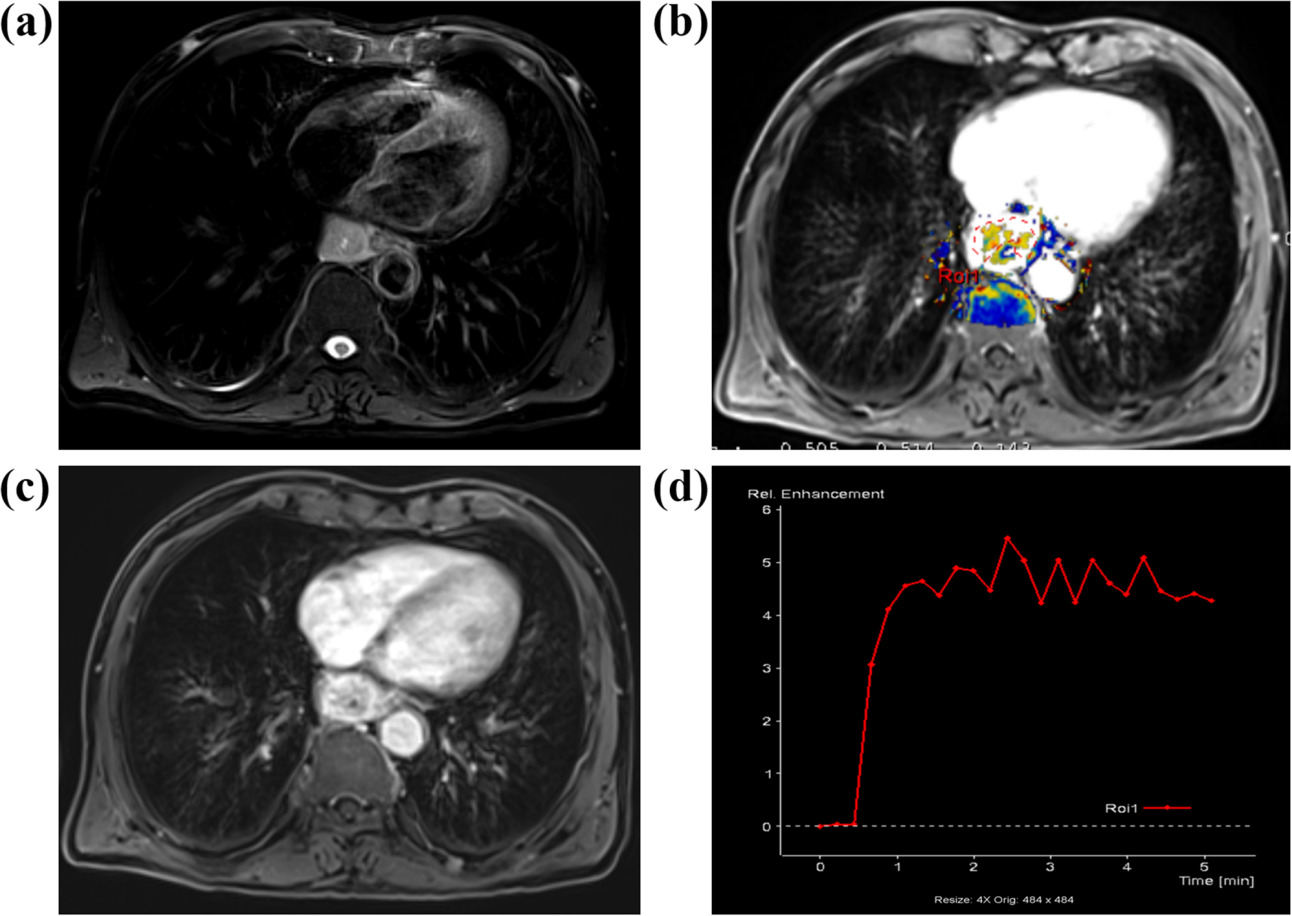
A patient (the age, 50–55) with esophageal carcinoma, perfusion scanning was performed with conventional 3D-VIBE sequence. (**a**) Obvious thickening of the esophageal wall and stenosis of the lumen was shown in the axial T2 fat depression image. (**b**) The pseudo-color map of Ktrans image. (**c**) The 13th stage image of the perfusion images. The inner and outer contours of the lesion are satisfactory, and the respiratory artifact is obvious. (**d**) The dynamic enhancement curve of region of interest (ROI 1) shows fast-rising-platform characteristics; and the perfusion parameters are Ktrans 0.76 min^−1^, Kep 1.18 min^−1^ and Ve 0.66.

**Figure 2:**
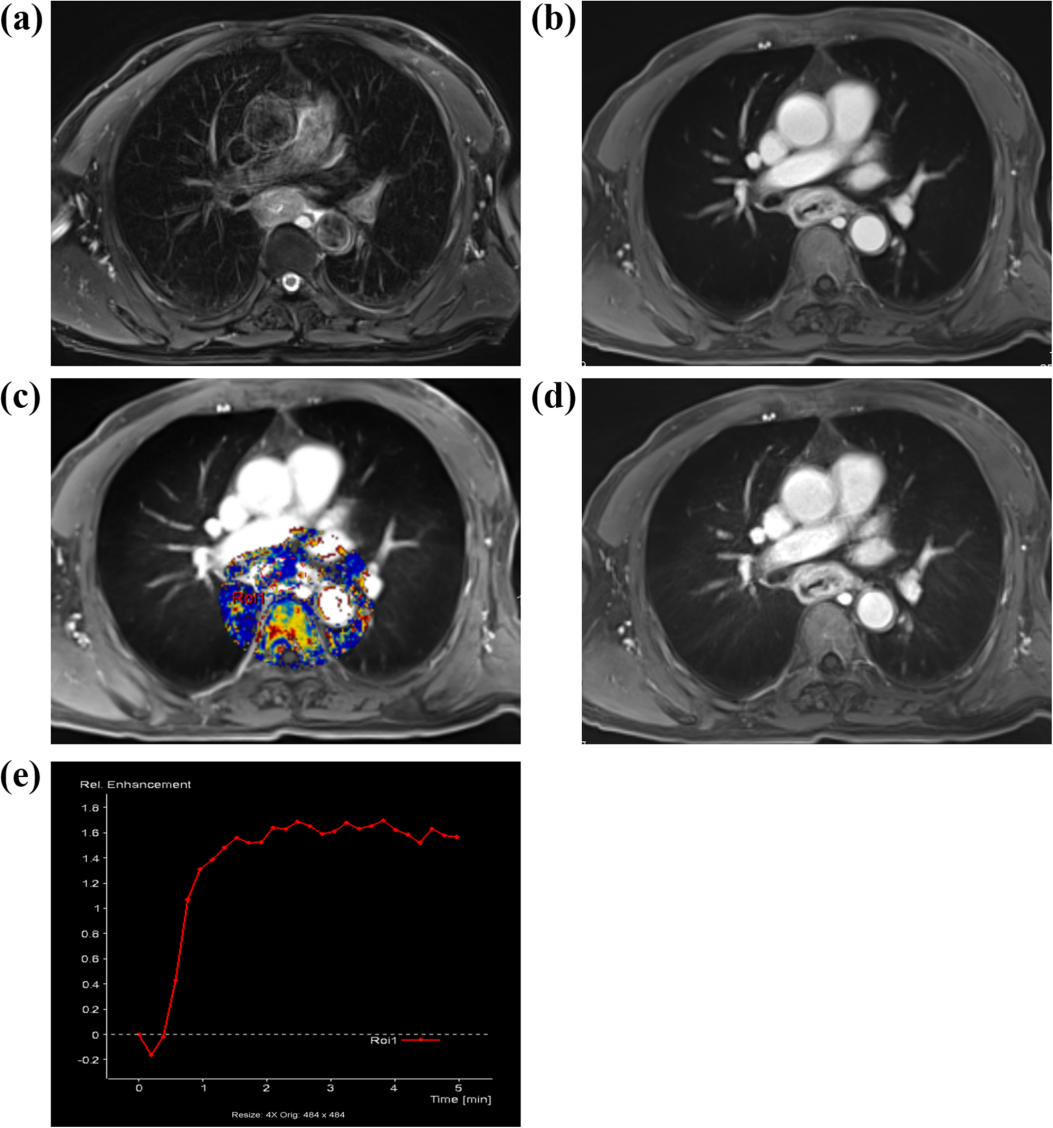
A patient (the age, 55–60) with esophageal carcinoma, perfusion scanning was performed with star-VIBE sequence. (**a**) The axial image of T_2_ fat suppression shows thickened esophageal wall and narrowed lumen. (**b**) No respiratory artifact was demonstrated in the constructed axial image acquired with T1 fat suppression star-vibe sequence. (**c**) The pseudo-color map of Ktrans in interest regional of volume (Vol), in which ROI 1 is the lesion region. (**d**) The 14th phase image with original star-VIBE sequence data shows satisfactory inner and outer contours of the lesion, and the respiratory artifact was not obvious. (**e**) The dynamic enhancement curve of ROI 1 shows a quick rise platform characteristic, and the perfusion parameters are Ktrans 0.51 min^−1^, Kep 0.89 min^−1^, Ve 0.58.

The image quality of group A was better than that of group B, as supported by the higher average rank of group A than that of group B (35.90 vs 25.10, *p* = 0.009) (Table 1), as shown in Figs. 1c and 2d.

**Table 1:** Comparison of subjective image quality grading between the groups.

Group	Number of cases	Average rank	*Z*	*P*
A	30	35.90	− 2.623	0.009
B	30	25.10

### Comparison of SNR, CNR, Ktrans, Kep and Ve between the groups

The SNR, CNR and DCE-MRI parameters of the two groups are shown in Table 2. The SNR and CNR of group A were significantly higher than those of group B (*p* = 0.004 and < 0.001, respectively). There was no significant difference in Ktrans, Kep and Ve between the two groups (all *p* > 0.05) (Figs. 1, 2).

**Table 2:** Comparisons of objective image quality and DCE-MRI parameters between the groups.

Parameters	Group A	Group B	*t*	*P*
SNR	77.52 ± 20.91	58.63 ± 28.07	2.957	0.004
CNR	54.59 ± 15.92	36.45 ± 19.28	3.974	0.000
Ktrans (min^−1^)	0.61 ± 0.58	0.55 ± 0.36	0.441	0.661
Kep (min^−1^)	1.02 ± 0.89	0.85 ± 0.60	0.909	0.368
Ve	0.61 ± 0.14	0.68 ± 0.14	− 1.703	0.094

*SNR* signal to noise ratio, *CNR* contrast noise ratio, *Ktrans* volume transport constant, *Kep* reflux rate constant, *Ve* vascular extracellular volume fraction.

### Intraobserver and interobserver variability of image quality grading and DCE-MRI parameters

As demonstrated in Table 3, there was excellent intra- and interobserver variability in the measurement of Kep (ICC = 0.900–0.984 and 0.934–0.989, respectively) and moderate intra- and interobserver variability in the measurement of image quality grading (ICC = 0.820–0.932 and 0.724–0.901, respectively), Ktrans (ICC = 0.732–0.953 and 0.710–0.948, respectively) and Ve (ICC = 0.595–0.923 and 0.512–0.904, respectively).

**Table 3:** Intra- and inter-observer variability of image quality grading and perfusion parameters.

	Intra-observer		Inter-observer	
	ICC	95% CI	ICC	95% CI
Image quality grading	0.888	0.820–0.932	0.835	0.724–0.901
Ktrans	0.885	0.732–0.953	0.874	0.710–0.948
Kep	0.959	0.900–0.984	0.973	0.934–0.989
Ve	0.817	0.595–0.923	0.775	0.512–0.904

*ICC* intraclass correlation coefficient; *CI* confidence interval.

## Discussion

Our results found that without holding breath, the star-VIBE sequence's image quality is better than 3D-VIBE (SNR *p* = 0.004 and CNR *p* < 0.001, respectively). However, there was no significant difference in Ktrans, Kep or Ve between the groups (all *p* > 0.05), which proved the difference in filling methods of K-space did not affect the perfusion results. So, we can apply star-VIBE sequence to evaluating acquire the perfusion parameters (Ktrans, Kep and Ve) of tumors, which can noninvasively quantify tumor growth characteristics and vascular permeability^9,10^, further guiding diagnosis, staging, treatment and efficacy evaluation^11,12^.

Currently, common DCE-MRI sequence uses the 3D volume interpolated breath-hold examination (3D- VIBE) T_1_WI sequence. Due to its linear K-space filling method, it is sensitive to physiological movements such as respiration and easily produces motion artifacts in images^13^. To improve the image quality, the patients are required to hold their breath to suppress respiratory motion artifacts. However, prolonged breath holding is not feasible because there is a long period of continuous multiphase scanning in DCE-MRI scanning. At present, there are two common clinical methods to solve these problems. The first is holding the patient's breath at intervals of 3–5 s and then again in the perfusion examination^14^. However, the patients needed to cooperate to a high degree due to the high number of breath-holding times, and the perfusion condition of the lesion could not be obtained during the breathing interval between the two breath-holding scans. The scanning period is relatively small, which may affect the perfusion results. The second is breathing calmly in perfusion scanning and then using 3D nonrigid registration technology to reduce the influence of motion artifacts^15^. This technique is good for overcoming minor movements but has limited value for obvious respiratory artifacts. In this study, perfusion scanning with a routine conventional 3D-VIBE sequence was performed in group B under calm breathing, and the spatial position matching for lesions in each phase was poor, resulting in deviation in the region of interest during postperfusion processing and significant fluctuation in the signal values measured in the region of interest in the adjacent phase. The time-signal curve was changed in a zigzag pattern, and the fluctuation of the lesion signal at the same position in the adjacent phase was caused by respiratory movement.

The star-VIBE using K-space radial filling is insensitive to motion and can inhibit motion artifacts. At present, the images reconstructed from the star-VIBE sequence are the main clinical application^16^; however, few studies have used them to acquire raw data. Although the raw star-VIBE sequence data were used in the DCE-MRI scanning of gastric cancer by Li et al.^7^, the temporal resolution of each phase in the original star-VIBE sequence scanning data was not consistent. The temporal resolution was relatively high in the earlier phase, but it was significantly reduced in the later phase, as shown by the time-signal curve of gastric cancer perfusion^7^. In our study, some scanning parameters of the star-VIBE sequence were modified (subframe interval 2), the temporal resolution of the raw data was consistent, and the temporal resolution of each phase met the perfusion demand. We found that the SNR and CNR in group A were higher than those in group B, which was consistent with the research of Shin et al.^17^. At the same time, the subjectively evaluated image quality of group A was superior to that of group B. The conventional 3D-VIBE sequence used in Fig. 1c shows obvious respiratory artifacts and blurred lung textures in the image, which may have a certain influence on the display of esophageal lesions. However, the respiratory artifact was significantly reduced, and the lung texture and esophageal lesions were better demonstrated when the star-VIBE sequence was used, as shown in Fig. 2d.

Through the subjective and objective analysis of the images of conventional 3D-VIBE and star-VIBE sequences, it was shown that the image quality obtained by the star-VIBE sequence was superior to that obtained by the conventional 3D-VIBE sequence under the condition of free breathing. In this study, perfusion analysis was conducted in the two groups at the same time, and there was no significant difference in the obtained main perfusion parameters (Ktrans, kep and Ve), which may indicate that the linear K-space filling pattern of the conventional 3D-VIBE sequence had no effect on the accurate judgment of tumor perfusion results compared to the star-VIBE sequence with radial K-space filling. The star-VIBE sequence using the radial K-space filling method was insensitive to respiratory movement^6,7,18^; thus, the degree of spatial matching of images in each phase was good, and the obtained time signal curve was more stable and accurate than the conventional 3D-VIBE sequence, as shown in our study. Furthermore, a new technology of GRASP (Golden-angle Radial Sparse Parallel) using continuous golden angle star-VIBE scan was developed, which can improve temporal resolution and further decrease motion artifact.

There are some limitations to this study. First, it was a single-center study with a relatively small sample, and a multicenter study with a larger sample is needed to validate our results. Second, the sequence is designed to use its reconstructed image but not original data; therefore, it is impossible to arbitrarily increase or decrease the scanning phase and change the temporal resolution of each phase. Third, the lesion display is poor in some cases of upper esophageal carcinoma due to magnetic sensitive artifacts, as shown in conventional 3D-VIBE sequences.

In conclusion, the star-VIBE sequence can be used in the perfusion examination of esophageal carcinoma, which is more advantageous than the conventional 3D-VIBE sequence in a free breathing state.
